# Hierarchically Structured Graphene Aerogel Supported Nickel–Cobalt Oxide Nanowires as an Efficient Electrocatalyst for Oxygen Evolution Reaction

**DOI:** 10.3390/molecules29081805

**Published:** 2024-04-16

**Authors:** Donglei Guo, Jiaqi Xu, Guilong Liu, Xu Yu

**Affiliations:** 1 Key Laboratory of Function-Oriented Porous Materials, College of Chemistry and Chemical Engineering, Luoyang Normal University, Luoyang 471934, China; gdl0594@163.com (D.G.); 18836032666@163.com (J.X.);; 2 Institute of Innovation Materials and Energy, School of Chemistry and Chemical Engineering, Yangzhou University, Yangzhou 225002, China

**Keywords:** hierarchical structure, nickel-cobalt oxide, graphene aerogel, oxygen evolution, transition metal nanowire

## Abstract

The rational design of a heterostructure electrocatalyst is an attractive strategy to produce hydrogen energy by electrochemical water splitting. Herein, we have constructed hierarchically structured architectures by immobilizing nickel–cobalt oxide nanowires on/beneath the surface of reduced graphene aerogels (NiCoO_2_/rGAs) through solvent–thermal and activation treatments. The morphological structure of NiCoO_2_/rGAs was characterized by microscopic analysis, and the porous structure not only accelerates the electrolyte ion diffusion but also prevents the agglomeration of NiCoO_2_ nanowires, which is favorable to expose the large surface area and active sites. As further confirmed by the spectroscopic analysis, the tuned surface chemical state can boost the catalytic active sites to show the improved oxygen evolution reaction performance in alkaline electrolytes. Due to the synergistic effect of morphology and composition effect, NiCoO_2_/rGAs show the overpotential of 258 mV at the current density of 10 mA cm^−2^. Meanwhile, the small values of the Tafel slope and charge transfer resistance imply that NiCoO_2_/rGAs own fast kinetic behavior during the OER test. The overlap of CV curves at the initial and 1001st cycles and almost no change in current density after the chronoamperometric (CA) test for 10 h confirm that NiCoO_2_/rGAs own exceptional catalytic stability in a 1 M KOH electrolyte. This work provides a promising way to fabricate the hierarchically structured nanomaterials as efficient electrocatalysts for hydrogen production.

## 1. Introduction

Hydrogen energy with the merit of being a carbon-free product has become the focus of modern global attention and is considered the potential energy source to replace the consumption of transitional fossil fuels [1,2]. Hydrogen plays critical roles in both living organisms and various industrial processes, and the demand for hydrogen energy dramatically increased from 2000 (~59 Mt) to 2020 (~88 Mt), and is predicted to continue to rise by 2050 (~528 Mt) [3]. Electrochemical water splitting, including two half-reactions of the oxygen evolution reaction (OER) and hydrogen evolution reaction (HER), has attracted attention for producing hydrogen gas. Especially, the OER electrolysis is deemed to be a promising strategy for the formation of clean energy and energy conversion [4,5,6]. However, it is emergent to dissolve the issue of sluggish kinetics and complicated reactions during the OER process, which determines its faradic efficiency and requires a high overpotential to satisfy the practical applications [7]. The efficient electrocatalyst is the key factor in determining the catalytic reaction. The noble-metal-based electrocatalysts, such as iridium oxide (IrO_2_) and ruthenium dioxide (RuO_2_) [8,9,10], have been demonstrated to show outstanding OER activity, including low overpotential and high catalytic stability, but their high cost and lack resources directly hinder their widespread application in commercialization. Based on these features, it is important to develop a cost-effective and naturally sustainable catalyst for the OER process.

Transition-metal-based nanostructures (Fe, Co, Ni, etc.) with earth-abundant resources, low prices, and abundant surface-active sites are favored by researchers, including transition metal oxides/hydroxides (TMOs) [11,12], transition metal sulfides (TMSs) [13,14], transition metal phosphides (TMPs) [15,16], transition metal fluorides (TMFs) [17,18], and transition metal nitrides (TMNs) [19], which have been generally evaluated as appreciable OER catalysts. Among them, TMO-based nanostructured catalysts have attracted attention owing to their exposed surface area and active sites, and their hybrids can show modified surface chemistry to boost the catalytic active sites for improved faradic efficiency and catalytic stability [20]. It has been proved that the reaction intermediates with the existence of covalent metal-O bonds can accelerate the catalytic reaction, and the adjusted strength of metal-O bonds is studied as the active site [11,21]. Numerous synthetic methods have been applied to construct the controllable morphology of catalysts, which extraordinarily affects catalytic efficiency, such as one-dimensional nanorods and nanowires [22,23,24,25]. Furthermore, the metallic atom-doped TMO catalysts with the bi-metallic alloying mechanism can result in surface reconstruction and adjust the electronic structure to improve electrocatalytic performance [26]. Moreover, the poor electrical conductivity of catalysts caused by the agglomeration of nanocomposites can be further tuned by introducing the conductive matrix into the catalysts.

Apart from the chemical compositional features, the controllable morphological structure is also important to determine the OER activity of the catalyst [27,28]. Carbon-based nanomaterial with high electronic conductivity is a promising candidate to accelerate the charge transfer during the OER process, while the controllable morphology of carbon-based materials can be obtained from different synthetic methods [29,30,31,32], such as hydrothermal, thermal annealing, etc. Graphene oxides, containing affluent surface functional groups (–OH and –COOH, etc.), can be easily modified to construct the hierarchical structure by the self-assembly of graphene nanosheets, and the unique porous structure can expose the large surface area and provide pathways for electrolyte ion facilitation [33,34]. Furthermore, the hybridization of carbon materials with TM components forms a strong TM/carbon interface [33,35,36,37], which can dramatically increase the electronic conductivity of the catalyst to guarantee the long-term durability of a catalyst during the OER process. Therefore, hybridizing NiCo_2_ wires with hierarchical graphene aerogel as a cost-efficient catalyst for OER is an attractive strategy to satisfy the practical application of hydrogen production.

In this work, we have constructed the hierarchically structured electrocatalyst by hybridizing nickel–cobalt oxide nanowires with reduced graphene aerogels (NiCoO_2_/rGAs) through a facial hydrothermal method. The hierarchical structure remained after freeze-drying treatment, which is beneficial for providing a large surface area and increasing the utilization of active sites. The surface chemistry is tuned, and the surface-active sites are boosted by the strong electrochemical interaction at the NiCoO_2_/graphene interface. Due to the synergistic effect of high porosity and modified surface chemical states, NiCoO_2_/rGAs exhibit exceptional catalytic OER performance. To afford the current density of 10 mA cm^−2^, the overpotential of NiCoO_2_/rGAs is smaller with the lower Tafel slope and smaller charge transfer resistance than the control catalysts, implying a fast kinetic behavior for NiCoO_2_/rGAs. No apparent change in current density confirms good catalytic stability of NiCoO_2_/rGAs.

## 2. Results and Discussion

### 2.1. Morphological Structure of NiCoO_2_/rGAs

**Figure 1:**
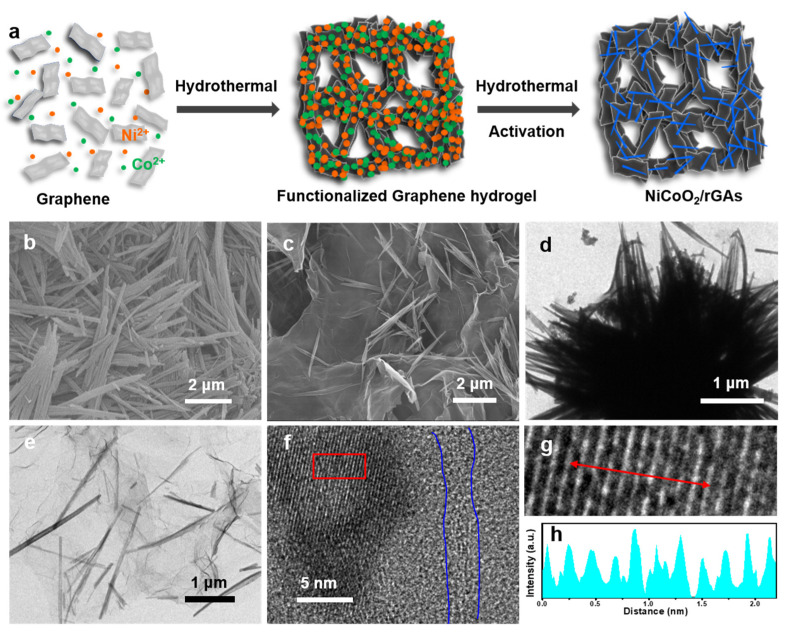
(**a**) Schematic illustration of the preparation of NiCoO_2_/rGAs. SEM images of (**b**) NiCoO_2_ and (**c**) NiCoO_2_/rGAs. TEM images of (**d**) NiCoO_2_ and (**e**) NiCoO_2_/rGAs. (**f**,**g**) High-resolution TEM images of NiCoO_2_/rGAs. (**h**) The measured lattice spacing of NiCoO_2_ in NiCoO_2_/rGAs (a profile of (**g**)).

A schematic illustration of the synthetic process of NiCoO_2_/rGAs is shown in Figure 1a, and the hierarchically structured catalysts are prepared through the hydrothermal method and low-temperature activation treatment. The quality of the mixture is crucial to prepare the hierarchically structured aerogel. Firstly, the nickel chlorides, cobalt chlorides, and graphene are mixed to obtain a uniform distribution and are treated via a facile hydrothermal method to initially obtain the metal-ion functionalized graphene hydrogel. During the hydrothermal process, graphene oxides with abundant surface functional groups (–OH, –COOH, etc.) are self-assembled by forming the π-π bonding configuration and hydrogen bonds, and the hierarchical structure of graphene hydrogel is constructed by the crosslink of graphene oxide nanosheets. Subsequently, the hydrogel was mixed with oxalic acid in ultrapure water, and the nanowires were grown on or beneath the surface of graphene, which is helpful to avoid the agglomeration of nanowires. After the freeze-drying treatment for 3 days, the aerogel with hierarchical structure is kept, which is further heated at 400 °C under a nitrogen atmosphere to finally obtain the NiCoO_2_/rGAs catalyst.

The morphological structure of NiCoO_2_/rGAs was initially probed by a scanning electron microscope (SEM). Figure 1b shows the morphology of NiCoO_2_ nanowires, and an apparent agglomeration of nanowires can be observed. After incorporating graphene aerogels during the hydrothermal and activation process, NiCoO_2_/rGAs show the hierarchical structure in Figure 1c, which is composed of graphene nanosheets and NiCoO_2_ nanowires. Meanwhile, the NiCoO_2_ nanowires distribute on/beneath the surface of graphene without severe agglomeration, which arises from the supporter of graphene aerogel. The merit of unique morphology is beneficial to provide channels for fast electrolyte diffusion, expose the effective active sites, and increase their utilization. The transmission electron microscopy (TEM) technique was further characterized to evaluate the morphology of NiCoO_2_/rGAs. As shown in Figure 1d, the aggregated NiCoO_2_ nanowires are observed. Figure 1e shows the TEM image of NiCoO_2_/rGAs. The transparent graphene nanosheets of NiCoO_2_/rGAs can be observed, and NiCoO_2_ nanowires are distributed on the surface of graphene without agglomeration, which agrees with the SEM analysis. The high-resolution TEM images are shown in Figure 1f,g, and Figure 1h is an enlargement of the selected region in Figure 1f. NiCoO_2_/rGAs show an apparent lattice fringe with a d-spacing of 0.21 nm, corresponding to the (200) plane of NiCoO_2_ in Figure 1h [38]. Meanwhile, the region marked in blue is ascribed to graphene oxides.

**Figure 2:**
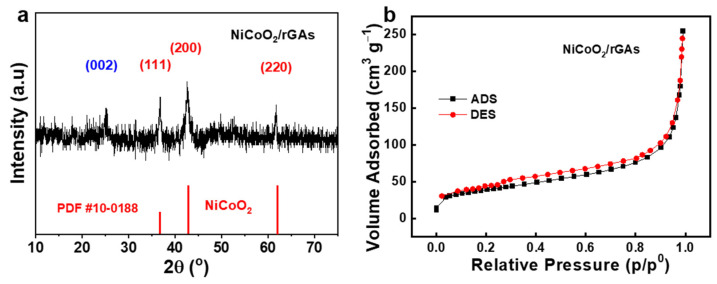
(**a**) XRD spectra and (**b**) nitrogen adsorption/desorption isotherms of NiCoO_2_/rGAs.

The high porosity of NiCoO_2_/rGAs was evaluated by the nitrogen adsorption/desorption isotherms in Figure 2a, and the specific surface area is 121.5 m^2^ g^−1^ with an average pore size of 14.3 nm. The large specific surface area is beneficial to increase the contact between the catalyst and electrolyte and expose the boosted active sites on the surface of the catalyst. To verify the crystallinity of NiCoO_2_/rGAs, an X-ray diffraction technique was carried out in Figure 2b. The diffraction peak for rGAs centering at 25.3° is indexed to the typical (002) pattern of graphite carbon, implying that the unstable surface functional groups on the surface of graphene are reduced [39]. In comparison to rGAs, NiCoO_2_/rGAs show a weak diffraction peak at 25.3° corresponding to the (002) plane of graphite carbon, and the slight shift of (002) peaks can be assigned to the expanded distance by forming the NiCoO_2_/graphene interface. Meanwhile, other XRD patterns of NiCoO_2_/rGAs are consistent with the standard NiCoO_2_ [PDF #10-0188], and three primary diffraction peaks indexed at 36.7°, 42.7°, and 61.8° are evident for the NiCoO_2_/rGAs and are assigned to the typical (111), (200), and (220) planes, respectively. The XRD analysis confirms that NiCoO_2_/rGAs consist of crystal NiCoO_2_ and graphene.

**Figure 3:**
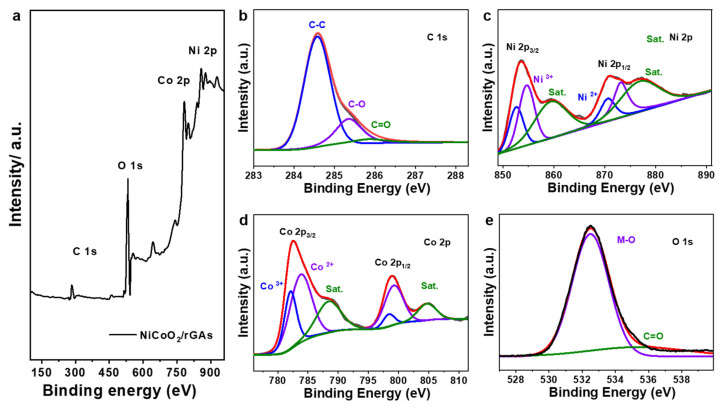
(**a**) Full survey XPS spectra and high-resolution (**b**) C 1 s, (**c**) Ni 2 p, (**d**) Co 2 p, and (**e**) O 1 s spectra of NiCoO_2_/rGAs.

To evaluate the surface environment of the NiCoO_2_/rGA catalyst, X-ray photoelectron spectroscopy (XPS) was carried out, and all the spectra were calibrated by C 1 s peaks at 284.6 eV. As shown in Figure 3a, the full scan XPS spectra show four primary peaks corresponding to the C, O, Co, and Ni elements, and the strong peak of the oxygen element may primarily come from the formation of bi-metallic oxides by decomposing the synthesized metal compounds and the adsorbed water during the sample preparation process. Figure 3b shows the high-resolution C 1 s spectra of NiCoO_2_/rGAs, and the deconvoluted peaks at 284.6, 285.3, and 285.9 eV are attributed to the C-C, C-O, and C=O bonds [29]. For the high-resolution Ni 2 p spectra in Figure 3c, two primary peaks at the binding energy of 853.5 and 870.9 eV correspond to the spin orbital of Ni 2 p_3/2_ and Ni 2 p_1/2_, accompanied by the related satellite peaks at 859.6 and 877.2 eV, respectively [40]. Meanwhile, the Ni 2 p_3/2_ can be rationally divided into two peaks at 852.6 and 854.7 eV owing to the Ni^2+^ and the multiple splitting of Ni^3+^, and two fitted peaks for Ni 2 p_1/2_ at 870.5 and 873.2 eV correspond to Ni^2+^ and of Ni^3+^, implying the presence of divalent oxidized species of Ni in NiCoO_2_/rGAs. The core-level spectra of Co 2 p are shown in Figure 3d, which consist of the two spin orbital doublets and the related shakeup satellite peaks. Four easily discernible peaks in Co 2 p can be fitted, including the major peak of Co 2 p_3/2_ with the related satellite peak and the primary peak of Co 2 p_1/2_ with the related satellite peak, respectively [41]. The peaks centered at 782.5 and 798.9 eV are attributed to the Co 2 p_3/2_ and Co 2 p_1/2_ accompanied by their satellite peaks at 788.6 and 804.8 eV. The energy gap between Co 2 p and its shakeup satellite peak is about 4.8 eV for Co^2+^ and 6.4 eV for Co^3+^, indicating the existence of the divalent oxidized state of Co species in NiCoO_2_/rGAs. For the high-resolution O 1 s peak in Figure 3e, the peak can be deconvoluted into two primary oxygen contributions. The peak centered at 532.6 eV corresponds to the typical metal-O bond [42], and the other peak centered at 535.8 eV is ascribed to the C-O/C=O bond of surface oxidized carbon species. As confirmed by the above XPS analysis, NiCoO_2_/rGAs include the metallic Co and Ni ions and C and O elements. The atomic ratios of Ni and Co elements are 5.85% and 6.03%, which are matched with the stoichiometry ratio of NiCoO_2_.

### 2.2. Electrocatalytic OER Performance

**Figure 4:**
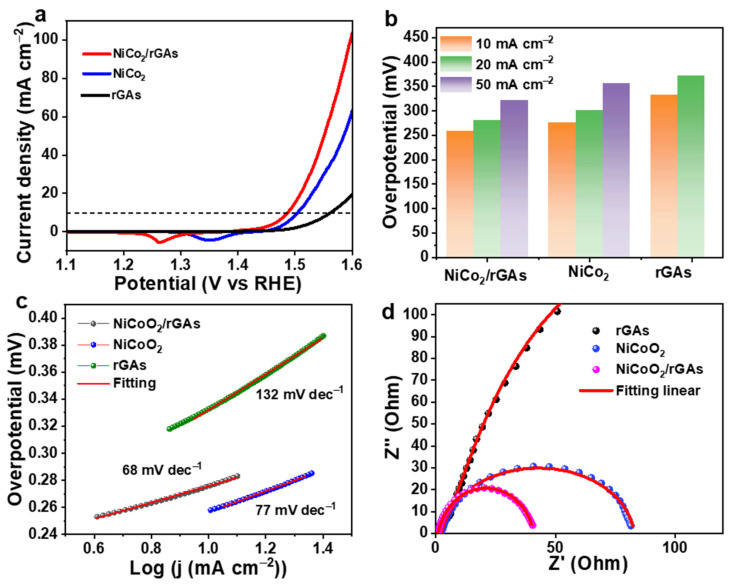
(**a**) Polarization curves at 5 mV s^−1^ in 1 M KOH, (**b**) the overpotentials at different current densities, (**c**) Tafel slopes, and (**d**) Nyquist plots of NiCoO_2_, rGAs and NiCoO_2_/rGAs.

To evaluate the electrochemical OER activity of NiCoO_2_/rGAs, a cyclic voltammetry (CV) test was applied through a typical three-electrode system, and 1 M KOH as the electrolyte was pretreated by constant flow of N_2_. After the activation of the catalyst, the polarization curve of NiCoO_2_/rGAs was measured at a scan rate of 5 mV s^−1^, and the control sample of NiCoO_2_ and rGAs catalysts was measured under identical conditions as the comparison. It is worth noting that all the potentials of catalysts have been calculated after IR correction. During the OER measurement, the NiCoO_2_/rGAs catalyst acts as the working electrode, and the graphitic carbon and saturated calomel electrode act as the counter and reference electrodes. The polarization curves of all catalysts at 5 mV s^−1^ are shown in Figure 4a, and NiCoO_2_/rGAs have an overpotential of 258 mV to afford a current density of 10 mA cm^−2^, which is more negative than that of NiCoO_2_ (275 mV) and rGAs (332 mV). Specifically, the overpotentials for NiCoO_2_/rGAs are 280 mV at the current density of 20 mA cm^−2^ and 321 mV at the current density of 50 mA cm^−2^, which are both lower than those of NiCoO_2_ (300 mV at 20 mA cm^−2^ and 355 at 50 mA cm^−2^) and rGAs (372 mV at 20 mA cm^−2^) in Figure 4b, unveiling that the NiCoO_2_/rGA as the non-noble-metal catalyst owns exceptional OER activity. Tafel slope is a crucial parameter to reflect the obstructive condition of the electrode during the reaction process, which can be obtained by the formula η = a + blog(j), where a and b are the constant values, η is the overpotential (mV), and j is the current density (mA cm^−2^). It is well known that a represents the overpotential when the current density is a unit value (1 A cm^−2^), which is strongly related to the intrinsic property and the surface chemical state of electrode materials [43], etc. The values of the Tafel slope are compared to evaluate the catalytic kinetics and calculated from their polarization curves. The values of Tafel slope are 68, 77, and 132 mV dec^−1^ for NiCoO_2_/rGAs, NiCoO_2,_ and rGAs in Figure 4c. A smaller Tafel slope for NiCoO_2_/rGAs elucidates a faster kinetic property and better catalytic OER activity than other control samples. As reported by lectures, a Tafel slope with values of 30, 40, and 120 mV dec^−1^ represents the Volmer process, the Volmer–Heyrovsky process, and the Heyrovsky process, which are the major rate-determining steps during the OER process [44]. NiCoO_2_/rGAs with a value of 68 mV dec^−1^ imply that the Volmer–Heyrovsky process is the dominant rate-determining step.

The dynamic property of NiCoO_2_/rGAs was further investigated by electrochemical impedance spectroscopy (EIS) in Figure 4d. The electrical equivalent circuit applied to deconvolute the Nyquist plots includes the parameters R_1_, R_s,_ and R_ct_, which are the reflections of solution resistance, contact resistance between electrode and electrolyte, and charge transfer resistance (inset of Figure 4d) [45]. At high frequency, the R_ct_ value of NiCoO_2_/rGAs is lower than that of NiCoO_2_ and rGAs with a semicircle with a smaller diameter, elucidating a fast charge transfer and exceptional OER activity for NiCoO_2_/rGAs during the OER process. The smallest Tafel slopes and R_ct_ values of NiCoO_2_/rGAs can be attributed to the introduction of graphene aerogel into the NiCoO_2_ system, and the electronic conductivity of NiCoO_2_/rGAs is dramatically increased, which can strongly guarantee the good electrocatalytic OER performance in an alkaline electrolyte.

**Figure 5:**
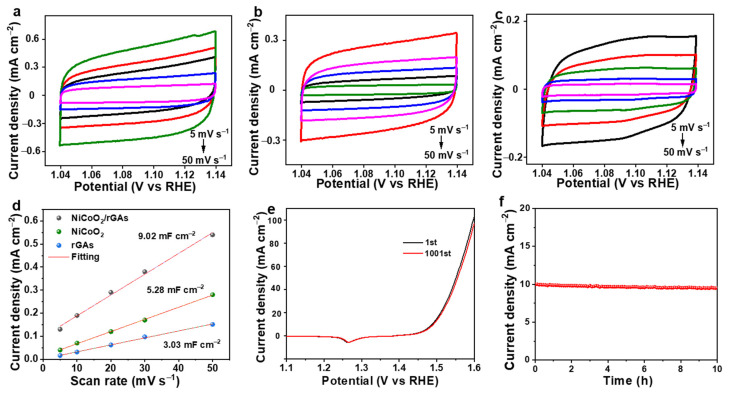
CV curves of (**a**) NiCoO_2_/rGAs, (**b**) NiCoO_2_, and (**c**) rGAs. (**d**) Cdl values of NiCoO_2_, rGAs and NiCoO_2_/rGAs. (**e**) CV curves at the 1st and 1001st cycles and (**f**) the CA test for 10 h of NiCoO_2_/rGAs.

NiCoO_2_/rGAs with a hierarchical structure can provide a large specific surface area and expose abundant active sites, which are favorable for improving electrocatalytic OER performance. To unveil the effect of hierarchical morphology on the catalytic OER performance, the double layer capacitance (C_dl_) was obtained by integrating the specific current density of CV curves. The CV curves of all the catalysts were tested at a series of scan rates from 5 mV s^−1^ to 50 mV s^−1^ (Figure 5a–c), and the calculated C_dl_ values for all catalysts are shown in Figure 5d. The C_dl_ value of NiCoO_2_/rGAs is 9.02 mF cm^−2^, which is about 1.7 and 3.0 times higher than that of NiCoO_2_ (5.28 mF cm^−2^) and rGAs (3.03 mF cm^−2^), implying a larger specific area and exposed active sites of NiCoO_2_/rGAs. The hierarchically structured graphene aerogel not only provides channels for fast ion diffusion but also inhibits the agglomeration of NiCoO_2_ nanowires and exposes the active sites for the OER test. To satisfy the practical application in hydrogen production, the long-term durability of the catalyst as a crucial factor was initially confirmed by CV curves for 1000 cycles at 5 mV s^−1^. In comparison to its initial cycle, the CV curve at the 1001st cycle is almost overlapped, and the overpotential is slightly increased with the value of 3 mV (Figure 5e), demonstrating that NiCoO_2_/rGAs owns good electrocatalytic OER durability. As further confirmed by chronoamperometry (CA) measurement at an overpotential of 258 mV for 10 h (Figure 5f), the current density has almost no loss during the sustaining operation. The CV curve for 1000 cycles and the CA test for 10 h strongly demonstrate the outstanding electrocatalytic OER stability in an alkaline electrolyte.

Due to the synergistic effect of the controllable morphological structure and composition, NiCoO_2_/rGAs can show exceptional OER performance in an alkaline electrolyte, and the detailed possibilities are listed below. (1) The hierarchical structure constructed from the self-assembly of graphene nanosheets can expose the large surface area and more active sites, accelerate the ion diffusion in the channels, and prevent the agglomeration of NiCoO_2_ nanowires. (2) The NiCoO_2_ nanowires with uniform morphology can deposit on or beneath graphene and act as the dominant active sites. (3) The strong chemical coupling between NiCoO_2_ and graphene can tune the surface chemical environment and boost the catalytic sites for the significant enhancement of OER performance.

## 3. Materials and Methods

### 3.1. Preparation of NiCoO_2_

A total of 10 mL of ultrapure water and 25 mL of ethylene glycol were mixed in a 100 mL glass beaker (100 mL) and then mixed with 150 mg of NiCl_2_·6H_2_O and 150 mg of CoCl_2_ under magnetic stirring for 20 min. Subsequently, 150 mg of oxalic acid was added into the beaker and stirred for 20 min. The dispersion was transferred to the stainless-steel autoclave (100 mL) and then the autoclave was heated at 180 °C for 10 h. The precipitation was washed with ethanol/deionized water several times and dried at 60 °C in a vacuum atmosphere. The powder was further annealed at 350 °C for 1 h under a nitrogen atmosphere with a heat rate of 10 °C min^−1^ and a gas flow rate of 100 cc min^−1^. The finally obtained sample was noted as NiCoO_2_ nanowires.

### 3.2. Preparation of rGAs

A total of 15 mL of graphene oxides (6 mg mL^−1^) were dispersed in 35 mL of ultrapure water under magnetic stirring for 20 min. The final dispersion was poured into a 100 mL stainless-steel autoclave and heated at 180 °C for 10 h. The hydrogel was washed by the solvent of ethanol/deionized water several times and solidified in liquid nitrogen. The hydrogel was freeze-dried for 3 days. The black aerogel was placed in the tube furnace and further annealed at 350 °C for 1 h at vacuum conditions. The sample was finally obtained and noted as rGAs.

### 3.3. Preparation of NiCoO_2_/rGAs

A mixture of ultrapure water (10 mL) and ethylene glycol (25 mL) was prepared in a glass beaker (100 mL) and worked as the solvent. A total of 150 mg of NiCl_2_·6H_2_O, 150 mg of CoCl_2,_ and 15 mL of graphene oxide dispersion (6 mg mL^−1^) were dispersed in the solvent under magnetic stirring for 20 min. Subsequently, 150 mg of oxalic acid was added into the beaker drop by drop with continuous magnetic stirring for 20 min to obtain the homogeneous dispersion. The final dispersion was poured into a 100 mL stainless-steel autoclave, and then the autoclave was heated at 180 °C for 10 h. The hydrogel was washed with the solvent of ethanol/deionized water several times and then immersed in liquid nitrogen until all the solvent was solidified. The solidified hydrogel was treated by freeze-drying for 3 days. The black aerogel was placed in the tube furnace and further annealed at 350 °C for 1 h at vacuum conditions. The heat rate is 10 °C min^−1^ and the flow rate of nitrogen gas is 100 cc min^−1^. The finally obtained sample was noted as NiCoO_2_/rGAs.

### 3.4. Characterization

The crystallinity of the catalyst was characterized by powder X-ray diffraction (XRD) (Bruker D8 Advance powder X-ray diffractometer, Cu Kα, λ = 1.5405 Å, 40 kV, Bruker, Billerica, MA, USA). Scanning electron microscopy (FESEM, Hitachi, S-4800 II, Tokyo, Japan) and transmission electron microscopy (TEM, Philips, TECNAI 12, Amsterdam, The Netherlands) were applied to evaluate the morphology. All X-ray photoelectron spectroscopy (XPS) measurements were probed to confirm the surface chemical state (Kratos XSAM-800 spectrometers, Al Kα radiation source, Manchester, UK).

### 3.5. Electrochemical Measurements

The electrochemical experiments were conducted using a DH7000C electrochemical workstation (Donghua Test, Taizhou, China) in a standard three-electrode configuration. The catalyst ink was prepared by mixing 5 mg of catalyst with 950 µL of ethanol and 50 µL of Nafion, followed by sonication for 30 min. The reference and counter electrodes consisted of a saturated calomel electrode (SCE) and a graphite rod, respectively. A glassy carbon electrode (GCE) was employed as the working electrode, and 10 µL of the catalyst ink was drop-cast and air-dried to achieve a loading of 0.71 mg cm^−2^. The potentials were calculated using the equation E(RHE) = E(SCE) + 0.0591 × pH + 0.242 V, with RHE representing the reversible hydrogen electrode.

Cyclic voltammetry (CV) measurements were performed across a range of current densities from 5 to 50 mV s^−1^, while the catalytic stability was evaluated through a chronoamperometry (CA) test conducted over a 10 h period. The kinetic properties of the catalyst were investigated using electrochemical impedance spectroscopy (EIS) over a frequency range from 100 kHz to 10 Hz. Additionally, CV curves were acquired in the non-faradic region at scan rates ranging from 5 to 50 mV s^−1^.

## 4. Conclusions

In summary, the hierarchically structured catalyst by coupling NiCoO_2_ nanowires with reduced graphene oxide aerogels was synthesized via a facile hydrothermal method and activation approach. As confirmed by microscopic analysis, the distribution of NiCoO_2_ nanowires on the surface of graphene is uniform, and the hierarchical structure of the catalyst can facilitate electrolyte diffusion and expose a large surface area. The surface chemistry of the catalyst is caused by the hybridization of nanowires with graphene, and the formation of an interface can boost the catalytic active sites. According to the specific morphology and tuned surface environment, NiCoO_2_/rGAs show outstanding OER performance, such as low overpotential, small Tafel slope and charge transfer resistance, and good catalytic durability. This study provides a straightforward methodology for fabricating hierarchically structured nanocomposites as efficient electrocatalysis for oxygen evolution reactions.

## Data Availability

Data are contained within the article.
